# Negative pressure pulmonary edema due to upper airway obstruction after general anesthesia in a patient with Parkinson's disease: A case report

**DOI:** 10.1002/ccr3.7444

**Published:** 2023-05-30

**Authors:** Ayaka Hasegawa, Naoko Niimi, Chieko Mitaka, Masakazu Hayashida

**Affiliations:** ^1^ Department of Anesthesiology and Pain Medicine Juntendo University Faculty of Medicine Tokyo Japan

**Keywords:** negative pressure pulmonary edema, Parkinson's disease, upper airway obstruction

## Abstract

In a patient with Parkinson's disease (PD) who underwent spine surgery 13 h after the last anti‐Parkinson medications, negative pressure pulmonary edema from upper airway obstruction developed immediately after extubation. Although oxygenation improved with high‐flow nasal cannula therapy, such complications might develop due to abrupt discontinuation of medication for PD.

## 1 BACKGROUND

Parkinson's disease (PD) is a common disease that is complicated by multiple respiratory abnormalities, including upper airway obstruction (UAO).^1^ Negative pressure pulmonary edema (NPPE) is a noncardiogenic pulmonary edema induced by the generation of high negative intrathoracic pressure needed to overcome UAO.^2^ To our knowledge, NPPE has not been reported in a patient with PD. We report the first case of NPPE that developed immediately after general anesthesia in a patient with PD.

## 2 CASE PRESENTATION

A 65‐year‐old man (175.0 cm, 65.0 kg) with a 23‐year history of PD and a 10‐year history of hypertension was scheduled to undergo C3–C7 cervical laminectomy with C3/C4 posterior fixation for cervical spondylosis. Preoperative examinations, including blood and urine tests, an electrocardiogram, and a chest X‐ray, showed no abnormal findings. The patient was taking oral medications for PD, including levodopa‐carbidopa (q.i.d). Symptoms of PD were well‐controlled so that he was able to live independently. He was admitted to the hospital the day before surgery. The patient took the last medications the night before surgery, 13 h before induction of anesthesia. Considering the well‐controlled PD status, anti‐Parkinson medications on the morning of surgery were neither administered orally nor replaced by those via alternative routes.

General anesthesia was induced and maintained with continuous infusions of propofol and remifentanil. Rocuronium (40 mg) was given to facilitate tracheal intubation. After the patient was placed in the prone position before surgery, neuromuscular relaxation was antagonized with sugammadex (150 mg) for monitoring of motor‐evoked potentials, which remained unsuppressed throughout surgery. Arterial oxygen saturation (SpO_2_) was maintained between 97% and 99%, and arterial oxygen tension (PaO_2_) was 201 mmHg with inspired oxygen fraction (F_I_O_2_) of 0.5. Fentanyl (150 μg) and acetaminophen (1000 mg) were intravenously administered for immediate postoperative analgesia. Durations of surgery and anesthesia were 161 and 254 min, respectively. Volumes of fluid infusion, blood loss, and urine output were 1410 mL, 60 mL, and 400 mL, respectively.

**FIGURE 1:**
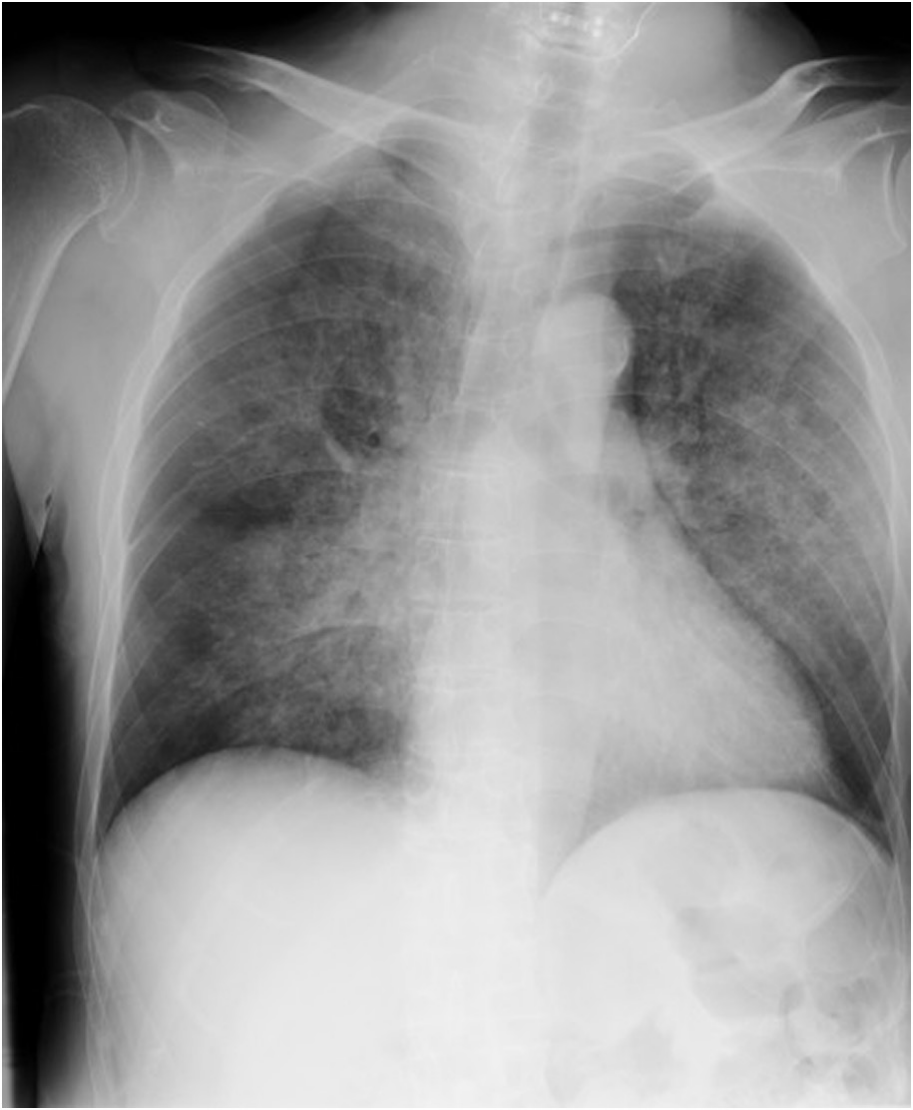
A chest X‐ray taken immediately after extubation showing diffuse bilateral pulmonary consolidation, consistent with negative pressure pulmonary edema.

After emergence from anesthesia, a sufficient tidal volume and respiratory rate were confirmed. The patient could take a deep breath and move extremities voluntarily, as requested by anesthesiologists. Immediately after tracheal extubation, however, the patient presented with labored breathing. Inspiratory stridor was heard. Systolic blood pressure exceeded 200 mmHg. SpO_2_ dropped to 78%. PaO_2_ was 61 mmHg during administration of oxygen at 8 L/min via face mask. The patient's UAO was immediately treated with a jaw thrust maneuver and tightly fitted face mask, which successfully improved signs and symptoms of UAO without a need for ventilatory support. An immediate chest X‐ray showed diffuse bilateral pulmonary consolidation without an increased cardiothoracic ratio, consistent with negative pressure pulmonary edema (NPPE) (Figure 1). Thus, 10‐cm H_2_O positive end‐expiratory pressure (PEEP) was applied to the spontaneously breathing patient using an anesthesia circuit. PaO_2_ increased to 105 mmHg.

The patient was transferred to the intensive care unit. High‐flow nasal cannula therapy (HFNC) was initiated with F_I_O_2_ of 0.6 and a flow rate of 50 L/min. NPPE improved by the morning of postoperative day (POD) 1; PaO_2_ increased to 204 mmHg with F_I_O_2_ 0.6. Oral anti‐Parkinson medications were resumed. On POD 2, the patient was weaned from HFNC, and returned to the ward without any oxygen supply.

## 3 DISCUSSION

Although UAO can develop as a manifestation of PD,^1^ it seemed necessary to rule out other causes of UAO in this patient. First, UAO due to alterations in the upper airway anatomy caused by cervical spine surgery^3^ could be ruled out because of no findings of upper airway stenosis on bidirectional cervical spine X‐rays taken before extubation. Second, respiratory depression and/or UAO due to effects of an anesthetic (propofol) and/or opioids (remifentanil and fentanyl) seemed negligible because the patient showed adequate respiration, alertness, and responsiveness before extubation. Third, residual neuromuscular block was unlikely, since the effect of low‐dose rocuronium (0.62 mg/kg) given 4 h before extubation was reversed with sugammadex before surgery for monitoring of motor‐evoked potentials, which remained unsuppressed throughout surgery. Lastly, laryngospasm, vocal cord paralysis, or upper airway edema could be ruled out, as just applying a jaw thrust maneuver resulted in an immediate improvement in signs and symptoms of UAO. Therefore, it was highly likely that UAO developed as a manifestation of PD,^1^ due to dystonia of oropharyngeal muscles, which is one of the three known etiologies of UAO in patients with PD, including bilateral vocal cords paralysis, laryngeal spasm, and dystonia of the oropharyngeal and neck muscles.^4^

The patient took the last anti‐Parkinson medications 13 h before anesthesia. The withdrawal of drugs might be long enough to diminish their effects, thereby causing UAO. Reportedly, duration of action of levodopa‐carbidopa to reduce motor disability lasts as short as 3–6 h.^5^, ^6^ After a 12‐h withdrawal of anti‐Parkinson medications, a significant change in the spirometry indicative of UAO occurs in a substantial number of PD patients,^7^, ^8^ which is reversed by levodopa.^7^ Especially, PD patients exhibiting laryngopharyngeal motor dysfunction, indicated mainly by hypophonia and impaired tongue protrusion, after a 12‐h drug withdrawal have a threefold greater chance of presenting with obstructive sleep apnea (OSA) unrelated to obesity, neck circumference, or the Mallampati score, compared with those without such dysfunction.^8^ Episodes of severe UAO that were successfully reversed by an anti‐Parkinson medication, including levodopa, have been reported.^9^, ^10^, ^11^ Such data suggest that in our patient, daily anti‐Parkinson medications should have been continued perioperatively to minimize the risk of developing UAO and subsequent NPPE.

According to a recent review, patients with PD are advised to have their anti‐Parkinson medications at fixed intervals daily to prevent fluctuations in motor and non‐motor symptoms of PD.^12^ Therefore, oral anti‐Parkinson medications should be administered as close as possible to the patient's medication schedule preoperatively and resumed at the earliest to diminish postoperative complications associated with PD, such as worsening of PD symptoms, aspiration pneumonia, delirium, falls, and even devastating dopamine withdrawal syndrome.^12^ If oral administration is difficult, alternative therapeutic options should be considered, such as carbidopa‐levodopa (sublingual), levodopa (oral inhalation), rotigotine (transdermal patch), and apomorphine (sublingual/subcutaneous injection/pump).^12^ Therefore, anti‐Parkinson medications should have been continued perioperatively in our patient.

In our patient, UAO and NPPE improved with HFNC without a resumption of anti‐Parkinson medications. HFNC is able to deliver a consistent oxygen supply to the alveoli by constantly applying positive pressure, enabling the patient to maintain a high level of oxygen supply.^13^ Further, generation of PEEP helps in reduction of anatomical dead space, carbon dioxide washout, recruitment of collapsed alveoli, and ultimate improvement in oxygenation.^13^ Through such effects, HFNC improves pulmonary edema.^13^, ^14^ In addition, HFNC improves UAO in patients with OSA and in patients under deep sedation with propofol.^15^, ^16^ Therefore, our experience also suggests that HFNC is effective in improving UAO and subsequent NPPE in patients with PD.

## 4 CONCLUSION

We experienced upper airway obstruction and negative pressure pulmonary edema that developed immediately after anesthesia and extubation in a patient with Parkinson's disease. Our experience suggests that in patients with Parkinson's disease, anti‐Parkinson medications should be continued perioperatively to minimize risks of such complications, and high‐flow nasal cannula therapy can improve upper airway obstruction and negative pressure pulmonary edema in such patients.

## AUTHOR CONTRIBUTIONS

**Ayaka Hasegawa:** Writing – original draft. **Naoko Niimi:** Writing – review and editing. **Chieko Mitaka:** Writing – review and editing. **Masakazu Hayashida:** Writing – review and editing.

## FUNDING INFORMATION

None.

## CONFLICT OF INTEREST STATEMENT

The authors declare that they have no competing interests.

## CONSENT

Written informed consent was obtained from the patient to publish this report in accordance with the journal's patient consent policy.

## Data Availability

The data that support the findings of this study are available from the corresponding author upon reasonable request.
